# Fishing down nutrients on coral reefs

**DOI:** 10.1038/ncomms12461

**Published:** 2016-08-16

**Authors:** Jacob E. Allgeier, Abel Valdivia, Courtney Cox, Craig A. Layman

**Affiliations:** 1 1122 NE Boat St. School of Aquatic and Fisheries Science, University of Washington, Seattle, Washington 98105, USA; 2 Oceans Program, Center for Biological Diversity, San Francisco, California 945612, USA; 3 Smithsonian Marine Station, Fort Pierce, Florida 34949, USA; 4 127 David Clark Labs, Department of Applied Ecology, North Carolina State University, Raleigh, North Carolina 27695, USA

## Abstract

Fishing is widely considered a leading cause of biodiversity loss in marine environments, but the potential effect on ecosystem processes, such as nutrient fluxes, is less explored. Here, we test how fishing on Caribbean coral reefs influences biodiversity and ecosystem functions provided by the fish community, that is, fish-mediated nutrient capacity. Specifically, we modelled five processes of nutrient storage (in biomass) and supply (via excretion) of nutrients, as well as a measure of their multifunctionality, onto 143 species of coral reef fishes across 110 coral reef fish communities. These communities span a gradient from extreme fishing pressure to protected areas with little to no fishing. We find that in fished sites fish-mediated nutrient capacity is reduced almost 50%, despite no substantial changes in the number of species. Instead, changes in community size and trophic structure were the primary cause of shifts in ecosystem function. These findings suggest that a broader perspective that incorporates predictable impacts of fishing pressure on ecosystem function is imperative for effective coral reef conservation and management.

## Abstract (web summary)

Coral reefs are productive ecosystems due to high levels of nutrient recycling in which fishes play a critical role. This study shows fishing can reduce the amount of nutrients supplied and stored by fishes to coral reefs by nearly half, even when the number of fish species present is largely unchanged.

## Graphical abstract

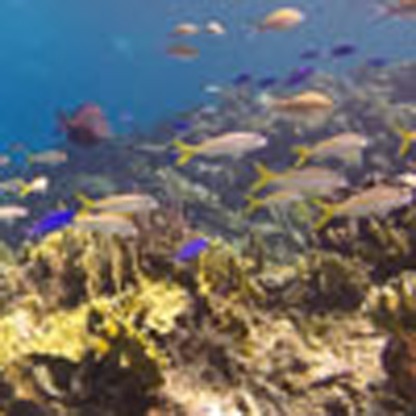

Biodiversity theory has provided important motivation for conservation efforts globally^1^. Theory largely has been supported by empirical data, but has often been limited to ecosystems with relatively few species and simplistic scenarios of community change, for example, random extinctions^2^. Yet many of the ecosystems most impacted by humans are among the most diverse, including tropical rainforests and coral reefs, and impacts are often non-random, disproportionately affecting certain aspects of communities^3^. While biodiversity conservation *per se* is imperative, additional human-driven changes to communities may necessitate additional conservation targets.

The productivity of many ecosystems depends on the nutrient capacity of the ecosystem; here defined as the total nutrients stored within, and the rate at which nutrients are recycled between the constituents of the system (the ambient nutrient availability, and the nutrients stored within plant and animal biomass)^4^,^5^. Coral reefs are replete with solar energy, but have low ambient nutrient availability and typically receive little sustained exogenous nutrient input (notable exceptions may be increased nutrient input associated with seasonal rains or upwelling)^5^,^6^. Thus, the high rates of production found within these ecosystems are largely attributed to the nutrients stored and cycled by living biomass^7^,^8^,^9^.

Fishes typically make up a substantial component of living biomass on coral reefs and represent an important reservoir of nutrients in these ecosystems^10^,^11^. The removal of biomass via fishing likely has substantial implications for the nutrient capacity of coral reefs because: (1) nutrients are removed from the system and (2) existing nutrients are shunted into alternative pathways and storage pools such as, invertebrates, macroalgae and microorganisms. Further, because exogenous nutrient inputs to reefs are sparse, the replacement rate of nutrients that are removed via fishing is likely slow^7^,^8^,^12^—a dynamic that is analogous to the disruption of nutrient cycles in tropical rainforests following intensive timber harvest^13^.

Fish-mediated nutrient capacity is determined by a complex combination of species-level traits, such as stoichiometry and metabolism, that scale with individual body size^14^. At the community level, fish nutrient capacity is determined by distinct aspects of community structure beyond biomass^11^,^15^. For instance, richness, evenness and size structure of species within the community drive variation in fish nutrient capacity across relatively unimpacted coastal fish communities in the Caribbean^15^. Yet, it is unknown how fishing pressure, particularly through selective exploitation of certain species, alters fish nutrient capacity of coral reef ecosystems.

Here we estimated the effects of fishing pressure on coral reef fish nutrient capacity in 110 fish communities on 43 Caribbean coral reefs (Supplementary Fig. 1). Pressure ranged from reefs with minimal fishing within relatively well-enforced no-take marine reserves, to heavily fished reefs with no protection where large predatory fish are mostly absent and overall size distribution of fish communities are relatively small (Supplementary Table 1). Our goals were to: (1) test how different aspects of fish community structure, including richness, evenness, trophic structure, size structure and biomass, influence fish nutrient capacity; (2) assess how fishing pressure alters fish nutrient capacity across a fishing gradient; and (3) identify the primary drivers of reduced fish nutrient capacity due to fishing pressure.

Our results support commonly assumed theoretical relationships between species richness and ecosystem function; however, when accounting for the biomass of the communities, this relationship is nonexistent. Fishing reduced fish-mediated nutrient processes by nearly half, but, contrary to expectations, reduction in nutrient processes was not due to species loss. Instead, changes in trophic and size structure of the fish community was the main driver in reduced ecosystem function. Species extirpation/extinction is a critical conservation concern, but our analysis suggests that efforts to preserve trophic groups and community size structure are also needed to maintain fish-mediated nutrient capacity in coral reef ecosystems.

## Results

### Study design

**Figure 1:**
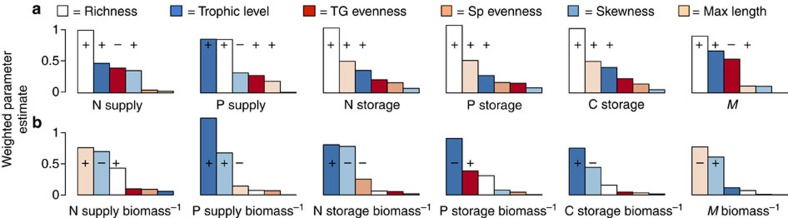
The relative importance of different aspects of community structure for fish nutrient capacity. (**a**) Bars indicate mean weighted parameter estimates (for models with ΔAIC<5) for mixed-effects model (with random intercepts for reef type and site nested within country; *n*=110) for six nutrient processes and *M* (multifunctionality). All models included six parameters: species richness (richness), species evenness (SD–reciprocal Simpson's index), Trophic Group evenness (TG), mean biomass-weighted trophic level (TL), mean maximum size per species (Max Length) and skewness of the size frequency distribution of the community (Skewness). Colours denote the different parameters of interest. ‘**+**' or ‘−' distinguish the direction of the response for parameters that were within the top model and for which the 95% confidence intervals do not overlap zero. (**b**) Mean weighted parameter estimates (for models with ΔAIC<5) for the same processes as in panel (**a**), but here corrected for relative biomass (*n*=110).

To understand the role of community structure for reef nutrient capacity, we tested the importance of biodiversity (e.g., richness and evenness) and other metrics of community structure (e.g., body size and trophic structure) in explaining nutrient processes using hierarchical mixed-effects models (Supplementary Methods; Fig. 1). Species richness and mean biomass-weighted trophic level of the community (trophic level: TL) were the best predictors of nutrient capacity (Fig. 1a). To disentangle the relative importance of biomass for all nutrient processes, we performed a second analysis with all response variables as biomass-specific processes. Thus, we were able to assess the relative importance of aspects of community structure beyond any inherent relationship with biomass. When accounting for biomass, richness was no longer an important predictor, but instead community size structure (skewness of size frequency distributions) and trophic structure (TL) emerged as the most important predictors (Fig. 1b).

These findings reveal two primary insights. First, they provide strong support for previous research^1^,^16^ by showing that the number of species within a community is a robust predictor of community-level nutrient processes (our models explained ∼77–82% of the variation in the data). Second, when considering the cumulative species-level effects on nutrient processes beyond the effect of biomass, the number of species within a community is a poor predictor (Fig. 1b,c). Instead, the most important attributes of a community for nutrient processes were the size distributions (skewness) and the TL of the communities (explaining ∼50% of the variation in the data). Notably, the direction of these relationships differed depending on the response variable of interest, for example, communities with more small individuals supplied higher rates of N but less P (Fig. 1b). This result highlights the differential importance of body size and diet, (metabolic theory and ecological stoichiometry theory, respectively), because smaller fish recycle nitrogen (N) at higher rates due to higher metabolism, but excrete less phosphorous (P) relative to larger fish due to a low P diet^14^,^17^.

### Fishing reduces nutrient capacity in coral reefs

**Figure 2:**
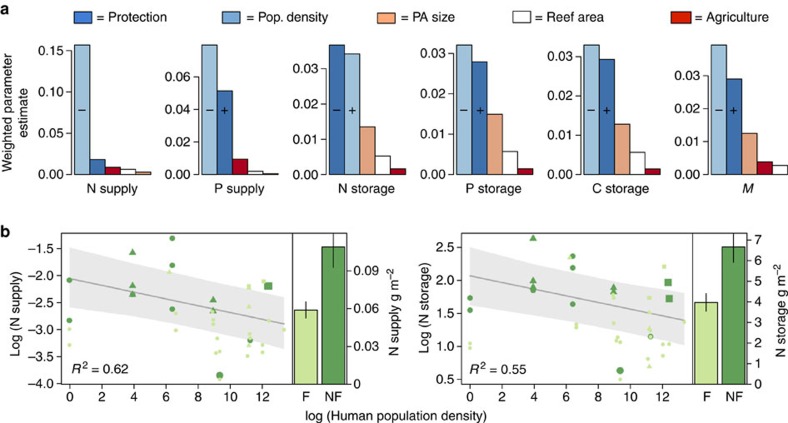
The relative importance of factors influencing fish nutrient capacity on reefs. (**a**) Bars indicate mean weighted parameter estimates for mixed-effects models (with random intercepts for reef type and country; *n*=39) for six ecosystem functions and *M* (multifunctionality). Predictor variables are arranged by colour and include: protection status–either fished or enforced protection (Protection; dark blue), distance to the nearest population settlement (Pop. Density; light blue), size of marine protected area (PA area; peach), total size of reef tract within 5 km radius of the surveyed site (Reef area; white) and area of cultivated land within 50 km–a proxy for terrestrial runoff (Agriculture; red). Colours denote the different parameters of interest. ‘**+**' or ‘−' distinguish the direction of the response for parameters that were within the top model and for which the 95% confidence intervals do not overlap zero. (**b**) Examples of relationships between N supply and storage and the most important continuous predictor–human population density (*n*=39). *R*^2^ values are for the mixed-effects model including only population density as a fixed effect. Grey bands indicate 95% confidence intervals. Symbols indicate different reef type (spur and grove=circle, wall=triangle and slope=diamond) and symbol size indicates relative size of protected area. Colours in all plots indicate protection status (light green=fished, dark green=protected). Barplots show differences between reefs that experience fishing (F) and are protected (NF)–all differences are significant. Error bars indicate s.e.

The next step in our analysis was to understand how humans mediate ecosystem function and the drivers associated with this change. Nutrient capacity mediated by coral reef fishes was substantially decreased by fishing and lack of protection (Fig. 2). This finding was determined by using mixed-effects models for all nutrient processes (responses) and five predictor variables associated with fishing and environmental characteristics of the system (Supplementary Methods; Fig. 2). A continuous measure of human population density (a common proxy of human impacts, in particular, fishing)^18^, and a categorical measure of protection status (here either: (i) no-take marine reserves with age 18–42 years, or (ii) areas open to fishing) were the most important predictors and were included in all top models (with the exception of N supply for which only human population density was included; Fig. 2; Supplementary Table 4). This difference is particularly notable when comparing fished and protected reefs, whereby fishing pressure reduced nutrient capacity 40–46% (Fig. 2).

**Figure 3:**
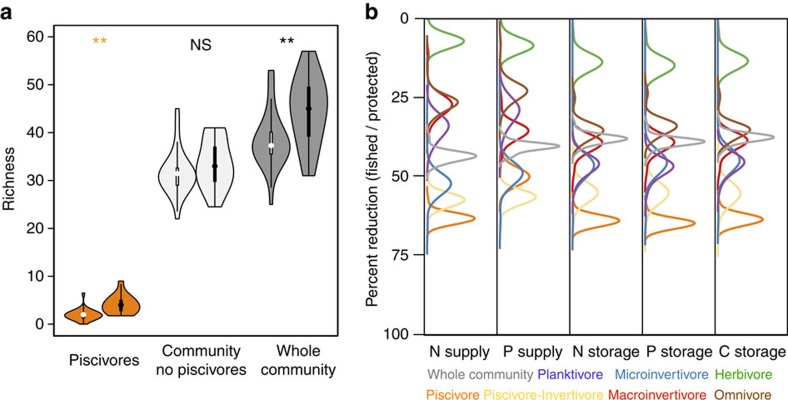
Effects of fishing on species richness and contribution of trophic groups for nutrient supply and storage. (**a**) The number of species lost due to fishing in various components of the community: piscivores only, the community minus piscivores and the whole community. Violin bars with a white centre bar indicate reefs on which fishing occurs versus a black centre bar indicating protected reefs (*n*=39 total communities). ‘**' and ‘NS' indicate statistical significance, and lack of significance, respectively, as determined by 95% confidence intervals overlap with zero from mixed-effects models. (**b**) Distributions of effect sizes illustrating the relative degree to which fishing reduces the supply and storage of nutrients by different trophic groups. Distributions represent bootstrapped (1000 iterations) per cent reductions in nutrient processes for each trophic group (*n*=7) between fished and protected reefs (*n*=39) (Supplemental Information: Human impacts analysis).

Identifying the drivers through which fishing reduces nutrient capacity in coral reefs is critical for informing appropriate fisheries management strategies to maintain ecosystem functions. We tested the effect of fishing pressure (human population density) on the two best predictors of nutrient capacity, TL and Richness, as well as on Biomass. Human population density was associated with substantially lower community TL than Biomass and Richness (Table 1) and explained more of the variation in community TL and Biomass than Richness—the same trends were found when using the categorical variable of fished and protected reefs (Supplementary Table 4). This finding supports previous assertions that fishing in coral reefs targets particular species, often those with higher trophic position that are generally larger^19^,^20^. Proportionately small differences in species richness were found between fished and protected reefs across the exploitation gradient (mean 39±6.0 s.d. versus 45±7.2 s.d. species, respectively; Fig. 3a). Piscivores, piscivore-invertivores and planktivores were the only trophic groups that showed significant reductions in richness (herbivores slightly increased) between fished and protected sites (7–4.9, 4.1–2, 2.5–2, species respectively; Supplementary Table 5). However, strong reductions in nutrient processes were found across all trophic groups between fished and protected reefs despite only minor reductions in richness (Fig. 3b). These findings underscore the disproportionate influence of altered size and trophic structure, not species loss, for nutrient capacity in the context of fishing pressure.

## Discussion

One mechanism by which species richness could be maintained despite selective fishing is through functional redundancy, whereby high levels of richness within trophic groups distributes the effect of fishing pressure across species^1^,^15^,^21^. Biodiversity theory predicts that high levels of functional redundancy should also help sustain high levels of ecosystem function^22^. However, across the fishing gradient, reduction in richness within a trophic (functional) group was not correlated with reductions in any process by that trophic group, with the exception of N supply, suggesting certain species disproportionately influence ecosystem function (Supplementary Fig. 2). Ecosystem function cannot be maintained simply by the presence of high numbers of species, as many other changes occur in the structure of the communities with fishing pressure^23^,^24^.

Recent attention has been given to understand how changes in animal populations alter nutrient dynamics on large ecological scales^25^. We show that targeted fishing of higher trophic levels is reducing the capacity of coral reef fish communities to store and recycle nutrients by nearly half. Fish-mediated nutrients enhance coral growth^26^ and primary production^27^, and may regulate nutrient ratios at the ecosystem scale^11^. Our findings underscore the growing need to incorporate animal-mediated nutrient dynamics in models of ecosystem function, particularly in light of the rapid rate of exploitation of animal biomass throughout the world^3^. Improved models of nutrient dynamics on coral reefs will also enhance our understanding of the negative impacts of anthropogenic nutrients for coral reefs that is drastically needed for conservation and management.

We provide perspectives on one aspect of nutrient capacity in coral reef ecosystems, using a snapshot view of fish-mediated nutrients based on animal biomass. Yet more comprehensive models of ecosystem nutrient dynamics will require integration of additionally important biological (e.g., invertebrates, microorganisms) and physical (e.g., upwelling, currents) components, as well as temporal perspectives of nutrient processes over time (e.g., incorporating animal secondary production and movement behaviour).

Rebuilding coral reef fish communities is of critical importance for food security and the livelihood of billions of people^28^. Recent evidence has shown that reef fish biomass may take 35–60 years to recover from heavily depleted levels^29^. Inherent to the recovery of these communities is the recovery of nutrient capacity^5^,^8^. We suggest that in addition to well-acknowledged conservation targets such as biodiversity protection, a broader perspective that incorporates predictable impacts of fishing pressure on nutrient dynamics is imperative for effective coral reef conservation and management.

## Methods

Fish-mediated nutrient capacity was measured as: nutrient storage (via fish biomass for carbon—C, nitrogen—N, and phosphorus—P), nutrient supply (via fish excretion for N and P), and an aggregate measure of all processes, specified as multifunctionality (*M*)^15^,^30^. We were not able to estimate fish production, due to lack of robust data for most of the species in the study, but instead provide a snapshot in time of potential nutrient storage and recycling via fish biomass. Each process was modelled onto 72,824 individual fish in 43 fore-reefs (10–15 m deep) in five Caribbean countries surveyed for multiple years (110 surveys total; Supplementary Table 1). We used species-specific models to estimate nutrient storage, supply and multifunctionality (Supplementary Methods), and summed these processes within sites for estimates of community-level nutrient capacity. Surveys across all sites included a total of 154 reef fish species (excluding sharks, which constituted <2% of the biomass across all sites and ∼9% of biomass within the 23 sites in which they were found). Our biogeochemical models allowed us to estimate nutrient processes for 143 fish species, including >99% of all non-shark biomass found within surveys.

### Data availability

The data that support the findings of this study are included in the Supplementary Data file 1.

## Additional information

**How to cite this article:** Allgeier, J. E. *et al*. Fishing down nutrients on coral reefs. *Nat. Commun.* 7:12461 doi: 10.1038/ncomms12461 (2016).

## Supplementary Material

Supplementary Information Supplementary Figures 1-2, Supplementary Tables 1-6, Supplementary Methods and Supplementary References.

Supplementary Data 1 Nutrient storage and supply, and community data for each surveyed fish community used in this study. The data file includes the amount of carbon, nitrogen, and phosphorus supplied and stored by each surveyed fish community. Corresponding metrics of fish community composition (e.g., richness, evenness, etc.) and site attributes are also included. See key tab for detailed terminology of column headings.

## Figures and Tables

**Table 1:** Effects of fishing on the structure of fish communities.

	**PopDensity**	***R***^**2**^
TL	−0.93 (0.07)	0.47
Biomass	−0.70 (0.05)	0.46
Richness	−0.66 (0.05)	0.34

Mixed-effects models were used (with random intercepts on reef type and country; *n*=43) to assess the relative influence of fishing pressure (with the continuous variable of human population density, here ‘PopDensity', as a proxy) on trophic level (TL), biomass or richness of fish communities. Bootstrapped regressions (*n*=100) were used to generate estimates of the effect size (shown here) for these relationships. In all cases response variables were *z*-scored so relative effect sizes could be compared directly. Values in ‘( )' indicated s.d.s from the mean of the bootstrapped estimates. In all models 95% confidence intervals of parameter estimates do not overlap with zero.

## References

[b1] Loreau M. . Biodiversity and ecosystem functioning: current knowledge and future challenges. Science 294, 804–808 (2001). 11679658 10.1126/science.1064088

[b2] Duffy J. E. Why biodiversity is important to the functioning of real-world ecosystems. Front. Ecol. Environ. 7, 437–444 (2009).

[b3] Estes J. A. . Trophic downgrading of planet earth. Science 333, 301–306 (2011). 21764740 10.1126/science.1205106

[b4] DeAngelis D. L. Dynamics of Nutrient Cycling and Food Webs Chapman and Hall (1992).

[b5] Hatcher B. G. Coral reef primary productivity–a hierarchy of patterns and process. Trends Ecol. Evol. 5, 149–155 (1990). 21232343 10.1016/0169-5347(90)90221-X

[b6] Szmant A. M. Nutrient enrichment on coral reefs: is it a major cause of coral reef decline? Estuaries 25, 743–766 (2002).

[b7] Pomeroy L. R. The ocean's food web, a changing paradigm. Bioscience 24, 9 (1974).

[b8] Deangelis D. L. . Nutrient dynamics and food-web stability. Annu. Rev. Ecol. Syst. 20, 71–95 (1989).

[b9] Sorokin Y. in Coral Reef Ecology Vol. 102, 215–249Springer Berlin Heidelberg (1995).

[b10] Newman M. J. H., Paredes G. A., Sala E. & Jackson J. B. C. Structure of Caribbean coral reef communities across a large gradient of fish biomass. Ecol. Lett. 9, 1216–1227 (2006). 17040324 10.1111/j.1461-0248.2006.00976.x

[b11] Allgeier J. E., Layman C. A., Mumby P. J. & Rosemond A. D. Consistent nutrient storage and supply mediated by diverse fish communities in coral reef ecosystems. Glob. Chang. Biol. 20, 2459–2472 (2014). 24692262 10.1111/gcb.12566

[b12] Johannes R. E. . Metabolism of some coral reef communities–team study of nutrient and energy flux at Eniwetok. Bioscience 22, 541 (1972).

[b13] Tiessen H., Cuevas E. & Chacon P. The role of soil organic-matter in sustaining soil fertility. Nature 371, 783–785 (1994).

[b14] Allgeier J. E., Wenger S. J., Schindle D. E., Rosemond A. D. & Layman C. A. Metabolic theory and taxonomic identity predict nutrient cycling in a diverse food web. Proc. Natl Acad. Sci. USA 112, 2640–2647 (2015). 10.1073/pnas.1420819112 PMC4443305 25877152

[b15] Allgeier J. E., Layman C. A., Mumby P. J. & Rosemond A. D. Biogeochemical implications of biodiversity loss across regional gradients of coastal marine ecosystems. Ecol. Monogr. 85, 132 (2015).

[b16] Cardinale B. J. . Biodiversity loss and its impact on humanity. Nature 486, 59–67 (2012). 22678280 10.1038/nature11148

[b17] Schindler D. E. & Eby L. A. Stoichiometry of fishes and their prey: implications for nutrient recycling. Ecology 78, 1816–1831 (1997).

[b18] Ward-Paige C. A. . Large-scale absence of sharks on reefs in the Greater-Caribbean: a footprint of human pressures. PLoS ONE 5, e11968 (2010). 20700530 10.1371/journal.pone.0011968 PMC2916824

[b19] DeMartini E., Friedlander A., Sandin S. & Sala E. Differences in fish-assemblage structure between fished and unfished atolls in the northern Line Islands, central Pacific. Mar. Ecol. Prog. Ser. 365, 199–215 (2008).

[b20] Pauly D., Christensen V., Froese R. & Torres F. Fishing down marine food webs. Science 279, 860–863 (1998). 9452385 10.1126/science.279.5352.860

[b21] Bellwood D. R., Wainwright P. C., Fulton C. J. & Hoey A. S. Functional versatility supports coral reef biodiversity. Proc. R. Soc. B-Biol. Sci. 273, 101–107 (2006). 10.1098/rspb.2005.3276 PMC1560014 16519241

[b22] Yachi S. & Loreau M. Biodiversity and ecosystem productivity in a fluctuating environment: the insurance hypothesis. Proc. Natl Acad. Sci. USA 96, 1463–1468 (1999). 9990046 10.1073/pnas.96.4.1463 PMC15485

[b23] Mouillot D. . Functional over-redundancy and high functional vulnerability in global fish faunas on tropical reefs. Proc. Natl Acad. Sci. USA 111, 13757–13762 (2014). 25225388 10.1073/pnas.1317625111 PMC4183327

[b24] Bellwood D. R., Hoey A. S. & Choat J. H. Limited functional redundancy in high diversity systems: resilience and ecosystem function on coral reefs. Ecol. Lett. 6, 281–285 (2003).

[b25] Doughty C. E. . Global nutrient transport in a world of giants. Proc. Natl Acad. Sci. USA 113, 868–873 1502549112– (2015). 26504209 10.1073/pnas.1502549112 PMC4743783

[b26] Meyer J. L., Schultz E. T. & Helfman G. S. Fish schools–an asset to corals. Science 220, 1047–1049 (1983). 17754550 10.1126/science.220.4601.1047

[b27] Allgeier J. E., Yeager L. A. & Layman C. A. Consumers regulate nutrient limitation regimes and primary production in seagrass ecosystems. Ecology 94, 521–529 (2013). 23691670 10.1890/12-1122.1

[b28] Sale P. F. . Transforming management of tropical coastal seas to cope with challenges of the 21st century. Mar. Pollut. Bull. 85, 8–23 (2014). 24997002 10.1016/j.marpolbul.2014.06.005

[b29] MacNeil M. A. . Recovery potential of the world's coral reef fishes. Nature 520, 341–344 (2015). 25855298 10.1038/nature14358

[b30] Hector A. & Bagchi R. Biodiversity and ecosystem multifunctionality. Nature 448, 188–U6 (2007). 17625564 10.1038/nature05947

