# Supplementary material for: Fishing down nutrients on coral reefs
Source: Nat Commun. 2016 Aug 16;7:12461. doi: 10.1038/ncomms12461 (PMC4990701; doi:10.1038/ncomms12461)
Supplement: Supplementary Information — Supplementary Figures 1-2, Supplementary Tables 1-6, Supplementary Methods and Supplementary References. [file ncomms12461-s1.pdf]

Supplemental Figure 1 – Map of sites where surveys were conducted.
Map was created using ArcGIS version 10

Figure S1.

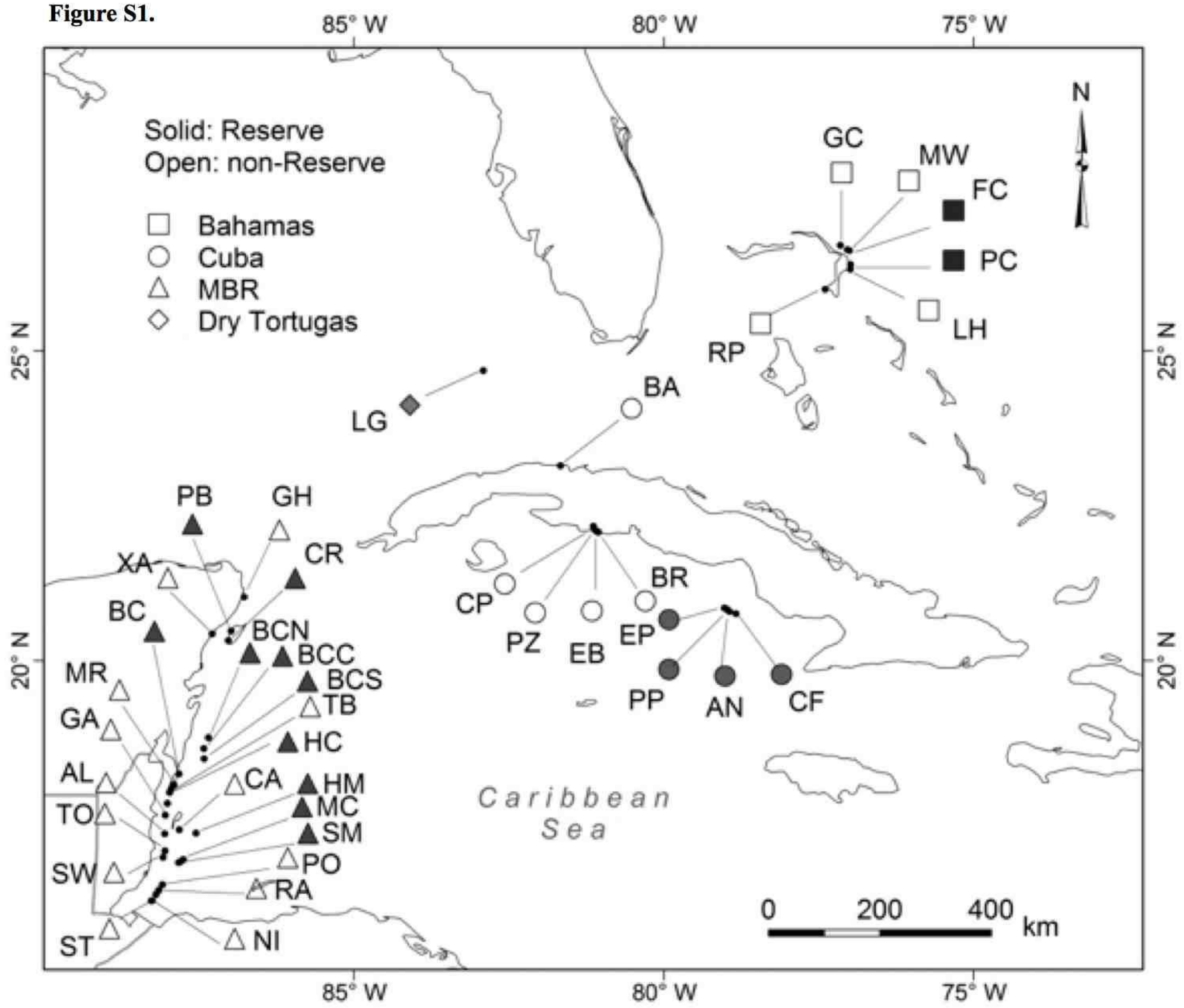

Supplemental Figure 2 – Proportional change in functional group richness and proportional change in nutrient process. Plots with no line indicate lack of statistical significance (p – value > 0.05).

Figure S2

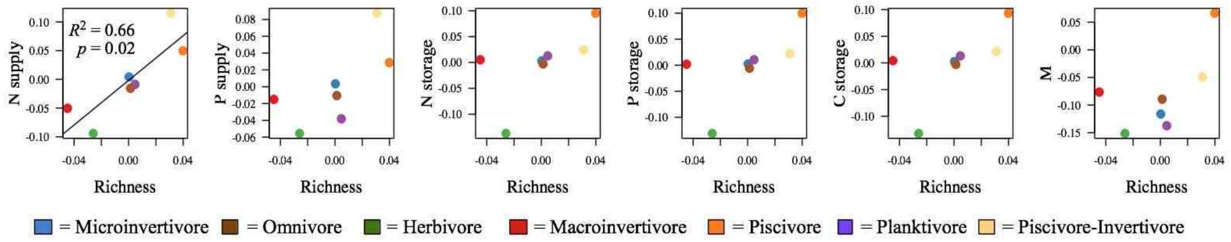

Supplemental Table 1 - Study sites, site codes, regions and protection level. Habitat type, S&G: Spur and Grove; Protection level, NTZ: No-take zone, MPA: marine protected area, GUA: general used area, NP: National Park. Date of survey is month and year(s).

Site name	Site code	Habitat type	Depth (m)	Date of survey	Latitude	Longitude	Protection level	MR year
Mesoamerican Barrier, Mexico								
Cancún	GH	S & G	12	Jul 12	21.02544	-86.7713	none	
Cozumel North	PB	S & G	10	Jul 12	20.47188	-86.9815	NTZ	1996
Akumal	XA	S & G	15	Jul 12	20.42689	-87.2860	none	
Cozumel South	CR	S & G	15	Jul 12	20.31961	-87.0266	NTZ	1996
Chinchorro North	BCN	S & G	15	Jul 12	18.74867	-87.3476	MPA	1996
Chinchorro Central	BCC	S & G	15	Jul 12	18.57457	-87.4198	MPA	1996
Chinchorro South	BCS	S & G	15	Jul 12	18.41008	-87.4169	MPA	1966
Mesoamerican Barrier, Belize								
Bacalar Chico	BC	S & G	12-15	May 10/12	18.16282	-87.82222	NTZ	1996
Mexico Rocks	MR	S & G	12-15	May 10/12	17.98782	-87.90382	none	
Tackle Box	TB	S & G	12-15	May 10/12	17.91056	-87.95083	none	
Hol Chan	HC	S & G	12-15	May 10/12	17.86343	-87.97238	NTZ	1987
Gallows	GA	S & G	12-15	May 10/12	17.49592	-88.04255	none	
Calabash Caye	CA	S & G	12-15	May 10/12	17.26147	-87.81970	none	
Half Moon Caye	HM	S & G	12-15	May 10/12	17.20560	-87.54679	NTZ	1982
Alligator Caye	AL	S & G	12-15	May 10/12	17.19660	-88.05115	none	
Tobacco Caye	TO	S & G	12-15	May 10/12	16.91911	-88.04757	none	
South Water Caye	SW	S & G	12-15	May 10/12	16.81346	-88.07756	MPA/GUA	1996
Middle Caye	MC	S & G	12-15	May 10/12	16.73703	-87.80536	NTZ	1993
South Middle Caye	SM	S & G	12-15	May 10/12	16.72875	-87.82867	NTZ	1993
Pampion Caye	PO	S & G	12-15	May 10/12	16.37310	-88.08913	none	
Ranguana Caye	RA	S & G	12-15	May 10/12	16.28501	-88.15031	none	
Southwest Caye	ST	S & G	12-15	May 10/12	16.11247	-88.27107	none	
Nicholas Caye	NI	S & G	12-15	May 10/12	16.11230	-88.25586	MPA/GUA	2003
Dry Tortugas, USA	LG	S & G	12	6/29/12	24.68508	-82.91050	NTZ	1992
Bay of Pigs, Cuba								
Cueva Peces	CP	Slope	10-12	Jun 11/12	22.16627	-81.13827	none	
Punta Perdiz	PZ	Slope	10-12	Jun 11/12	22.11003	-81.11626	none	
Ebano	EB	Slope	10-12	Jun 10	22.07914	-81.07599	none	
Brinco	BR	Slope	10-12	Jun 12	22.06939	-81.05588	none	
Bacunayagua, Cuba	BC	Slope	10-12	Jun 12	23.14653	-81.66664	none	
Jardines de la Reina, Cuba								
El Peruano	EP	Slope	10-12	Jun 11	20.84411	-79.02166	MPA/NP	1996
Pipin	PP	S & G	12-15	Jun 11	20.82586	-78.98026	MPA/NP	1996
Anclita	AN	Slope	10-12	Jun 11	20.78697	-78.94317	MPA/NP	1996
Cueva Pulpo	CF	Slope	10-12	Jun 11	20.75266	-78.83634	MPA/NP	1996
Abaco, Bahamas								
Guana Cay	GC	S & G	10-12	Jul 11/12	26.70967	-77.15408	none	
Fowls Cay	FC	Slope	10	Jul 11/12	26.63717	-77.03848	NTZ	2009
Man o' War	MW	S & G	10-12	Jul 11/12	26.62122	-77.00550	none	
Pelican Cay	PC	Slope	10	Jul 11/12	26.39783	-76.98850	NTZ	1972
Little Harbor	LH	S & G	10-12	Jul 11/12	26.32390	-76.99160	none	
Rocky Point	RP	Slope	10-12	Jul 11/12	25.99661	-77.40092	Remote	

Supplemental Table 2 – Mixed effects model results for biodiversity analysis.

N supply

int.	TG Ev.	Sp. Ev.	Lmax	Richness	Skew	TL	AIC	R^2 fixed	R^2 cond.	df	logLik	AICc	delta	weight
0.40	-0.42	0.00	0.00	0.96	0.34	0.48	154.69	0.54	0.81	9.00	-68.35	156.57	0.00	0.48
0.40	-0.36	-0.11	0.00	1.01	0.32	0.43	155.87	0.56	0.80	10.00	-67.94	158.19	1.62	0.22
0.40	-0.40	0.00	0.07	0.94	0.37	0.47	156.18	0.54	0.81	10.00	-68.09	158.50	1.93	0.18
0.40	-0.30	-0.16	0.12	1.00	0.36	0.40	156.59	0.56	0.81	11.00	-67.29	159.39	2.83	0.12

P supply

int.	TG Ev.	Sp. Ev.	Lmax	Richness	Skew	TL	AIC	R^2 fixed	R^2 cond.	df	logLik	AICc	delta	weight
0.38	-0.28	0.00	0.22	0.82	0.33	0.84	150.15	0.65	0.81	10.00	-65.08	152.47	0.00	0.57
0.38	-0.26	-0.02	0.22	0.82	0.33	0.83	152.13	0.65	0.81	11.00	-65.06	154.94	2.47	0.17
0.37	-0.33	0.00	0.00	0.87	0.24	0.86	153.30	0.65	0.80	9.00	-67.65	155.18	2.71	0.15
0.36	0.00	-0.19	0.31	0.85	0.36	0.71	154.51	0.66	0.80	10.00	-67.25	156.82	4.36	0.06
0.37	-0.38	0.08	0.00	0.84	0.26	0.89	154.86	0.65	0.80	10.00	-67.43	157.18	4.71	0.05

N storage

int.	TG Ev.	Sp. Ev.	Lmax	Richness	Skew	TL	AIC	R^2 fixed	R^2 cond.	df	logLik	AICc	delta	weight
0.51	-0.37	0.00	0.41	0.98	0.00	0.46	173.30	0.50	0.75	9.00	-77.65	175.17	0.00	0.23
0.50	-0.32	0.00	0.47	0.92	0.16	0.44	173.32	0.50	0.76	10.00	-76.66	175.64	0.46	0.18
0.51	-0.24	-0.19	0.47	1.04	0.00	0.37	173.47	0.52	0.75	10.00	-76.73	175.78	0.61	0.17
0.48	0.00	-0.31	0.59	1.00	0.18	0.27	174.06	0.52	0.75	10.00	-77.03	176.38	1.21	0.12
0.48	0.00	-0.36	0.54	1.08	0.00	0.25	174.58	0.52	0.74	9.00	-78.29	176.46	1.29	0.12
0.49	-0.21	-0.17	0.52	0.98	0.14	0.36	173.79	0.51	0.76	11.00	-75.90	176.60	1.43	0.11

P storage

int.	TG Ev.	Sp. Ev.	Lmax	Richness	Skew	TL	AIC	R^2 fixed	R^2 cond.	df	logLik	AICc	delta	weight
0.50	-0.32	0.00	0.42	1.02	0.00	0.40	172.28	0.50	0.75	9.00	-77.14	174.15	0.00	0.20
0.49	-0.27	0.00	0.48	0.96	0.16	0.39	172.19	0.50	0.76	10.00	-76.09	174.50	0.35	0.16
0.47	0.00	-0.27	0.58	1.02	0.18	0.23	172.68	0.52	0.76	10.00	-76.34	174.99	0.84	0.13
0.50	-0.21	-0.17	0.47	1.07	0.00	0.32	172.80	0.52	0.75	10.00	-76.40	175.12	0.97	0.12
0.48	0.00	-0.32	0.53	1.10	0.00	0.22	173.25	0.52	0.74	9.00	-77.62	175.12	0.97	0.12
0.49	-0.18	-0.15	0.52	1.00	0.15	0.31	173.00	0.51	0.76	11.00	-75.50	175.81	1.66	0.09

C storage

int.	TG Ev.	Sp. Ev.	Lmax	Richness	Skew	TL	AIC	R^2 fixed	R^2 cond.	df	logLik	AICc	delta	weight
0.50	-0.36	0.00	0.42	0.98	0.00	0.48	173.89	0.51	0.75	9.00	-77.94	175.76	0.00	0.28
0.50	-0.24	-0.18	0.48	1.03	0.00	0.39	174.26	0.52	0.75	10.00	-77.13	176.58	0.81	0.19
0.49	-0.32	0.00	0.47	0.93	0.13	0.47	174.50	0.51	0.76	10.00	-77.25	176.82	1.05	0.17
0.47	0.00	-0.35	0.55	1.07	0.00	0.28	175.35	0.53	0.74	9.00	-78.67	177.22	1.46	0.14
0.47	0.00	-0.30	0.59	1.00	0.15	0.29	175.48	0.53	0.75	10.00	-77.74	177.80	2.03	0.10
0.49	-0.21	-0.16	0.52	0.98	0.12	0.39	175.12	0.52	0.76	11.00	-76.56	177.93	2.16	0.10

M

int.	TG Ev.	Sp. Ev.	Lmax	Richness	Skew	TL	AIC	R^2 fixed	R^2 cond.	df	logLik	AICc	delta	weight
0.50	-0.48	0.00	0.21	0.83	0.22	0.64	172.89	0.50	0.77	10.00	-76.45	175.21	0.00	0.29
0.50	-0.57	0.00	0.00	0.93	0.00	0.66	174.47	0.51	0.74	8.00	-79.24	175.96	0.75	0.20
0.52	-0.55	0.00	0.14	0.91	0.00	0.65	174.62	0.51	0.75	9.00	-78.31	176.50	1.29	0.15
0.49	-0.53	0.00	0.00	0.88	0.13	0.66	174.94	0.51	0.75	9.00	-78.47	176.82	1.61	0.13
0.50	-0.46	-0.03	0.22	0.84	0.22	0.62	174.85	0.51	0.76	11.00	-76.43	177.66	2.46	0.08
0.50	-0.58	0.03	0.00	0.92	0.00	0.68	176.43	0.51	0.74	9.00	-79.22	178.31	3.10	0.06

Supplemental Table 3 – Mixed effects model results for biomass-corrected biodiversity analysis.

N supply	biomass^{-1}													
int.	TG Ev.	Sp. Ev.	Lmax	Richness	Skew	TL	AIC	R^2 fixed	R^2 cond.	df	logLik	AICc	delta	weight
0.05	0.00	-0.25	-0.66	0.47	0.60	0.00	260.37	0.35	0.49	9.00	-121.18	262.24	0.00	0.20
0.07	0.00	0.00	-0.68	0.40	0.67	0.00	260.82	0.35	0.46	8.00	-122.41	262.30	0.06	0.20
0.07	-0.24	0.00	-0.74	0.47	0.60	0.00	260.74	0.34	0.50	9.00	-121.37	262.62	0.38	0.17
0.04	-0.28	0.00	-0.76	0.39	0.58	0.19	261.63	0.34	0.53	10.00	-120.81	263.94	1.70	0.09
0.05	-0.14	-0.18	-0.70	0.49	0.58	0.00	261.84	0.35	0.50	10.00	-120.92	264.16	1.91	0.08
0.06	0.00	0.00	-0.69	0.35	0.67	0.11	262.44	0.35	0.47	9.00	-122.22	264.31	2.07	0.07

P supply	biomass^{-1}													
int.	TG Ev.	Sp. Ev.	Lmax	Richness	Skew	TL	AIC	R^2 fixed	R^2 cond.	df	logLik	AICc	delta	weight
0.07	0.00	0.00	-0.22	0.00	0.60	1.15	238.17	0.51	0.53	8.00	-111.09	239.66	0.00	0.15
0.06	0.00	0.00	0.00	0.00	0.65	1.13	238.56	0.50	0.52	7.00	-112.28	239.70	0.05	0.15
0.07	0.00	0.18	-0.24	0.00	0.62	1.17	238.53	0.52	0.54	9.00	-110.27	240.41	0.75	0.11
0.00	0.00	0.00	-0.23	0.23	0.54	1.06	238.67	0.53	0.53	9.00	-110.34	240.55	0.89	0.10
0.00	0.00	0.00	0.00	0.20	0.61	1.05	239.41	0.52	0.52	8.00	-111.71	240.90	1.24	0.08
0.07	0.00	0.14	0.00	0.00	0.68	1.14	239.52	0.51	0.52	8.00	-111.76	241.01	1.35	0.08

N storage	biomass^{-1}													
int.	TG Ev.	Sp. Ev.	Lmax	Richness	Skew	TL	AIC	R^2 fixed	R^2 cond.	df	logLik	AICc	delta	weight
0.23	0.00	-0.31	0.00	0.00	0.67	-0.75	272.25	0.23	0.52	8.00	-128.12	273.73	0.00	0.24
0.23	0.20	-0.43	0.00	0.00	0.69	-0.83	273.17	0.24	0.51	9.00	-127.59	275.05	1.31	0.12
0.26	0.00	0.00	0.00	0.00	0.71	-0.72	273.95	0.20	0.52	7.00	-129.97	275.09	1.36	0.12
0.26	0.00	0.00	0.00	-0.25	0.78	-0.61	274.18	0.21	0.52	8.00	-129.09	275.67	1.93	0.09
0.23	0.00	-0.27	0.00	-0.13	0.71	-0.69	273.85	0.23	0.52	9.00	-127.93	275.73	1.99	0.09
0.22	0.00	-0.32	0.05	0.00	0.69	-0.76	274.16	0.23	0.52	9.00	-128.08	276.04	2.31	0.08

P storage	biomass^{-1}													
int.	TG Ev.	Sp. Ev.	Lmax	Richness	Skew	TL	AIC	R^2 fixed	R^2 cond.	df	logLik	AICc	delta	weight
0.01	0.42	0.00	0.00	0.43	0.00	-0.91	298.05	0.16	0.22	8.00	-141.03	299.54	0.00	0.21
0.01	0.46	0.00	0.00	0.37	0.18	-0.93	299.27	0.16	0.23	9.00	-140.63	301.14	1.60	0.10
0.02	0.46	0.00	0.00	0.00	0.00	-0.70	300.16	0.13	0.21	7.00	-143.08	301.30	1.76	0.09
0.02	0.51	0.00	0.00	0.00	0.27	-0.78	300.17	0.14	0.23	8.00	-142.08	301.65	2.11	0.07
0.01	0.42	0.00	0.00	0.43	0.00	-0.91	300.05	0.16	0.22	9.00	-141.03	301.93	2.39	0.07
0.01	0.42	0.00	0.00	0.43	0.00	-0.91	300.05	0.16	0.22	9.00	-141.03	301.93	2.39	0.07

C storage	biomass^{-1}													
int.	TG Ev.	Sp. Ev.	Lmax	Richness	Skew	TL	AIC	R^2 fixed	R^2 cond.	df	logLik	AICc	delta	weight
-0.22	0.00	0.00	0.00	0.00	-0.49	0.62	281.73	0.12	0.47	7.00	-133.86	282.87	0.00	0.28
-0.21	0.00	0.00	0.00	-0.19	-0.44	0.70	282.66	0.13	0.47	8.00	-133.33	284.14	1.28	0.15
-0.21	-0.11	0.00	0.00	0.00	-0.51	0.65	283.29	0.12	0.47	8.00	-133.64	284.77	1.91	0.11
-0.22	0.00	0.00	0.06	0.00	-0.48	0.61	283.63	0.12	0.47	8.00	-133.81	285.11	2.24	0.09
-0.22	0.00	-0.02	0.00	0.00	-0.50	0.61	283.71	0.12	0.47	8.00	-133.85	285.19	2.32	0.09
-0.21	-0.08	0.00	0.00	-0.18	-0.46	0.72	284.43	0.13	0.47	9.00	-133.21	286.30	3.44	0.05

M	biomass^{-1}													
int.	TG Ev.	Sp. Ev.	Lmax	Richness	Skew	TL	AIC	R^2 fixed	R^2 cond.	df	logLik	AICc	delta	weight
-0.14	0.00	0.00	-0.73	0.00	0.51	0.00	279.20	0.19	0.51	7.00	-132.60	280.34	0.00	0.20
-0.08	0.00	0.00	-0.70	0.00	0.55	-0.25	278.98	0.20	0.51	8.00	-131.49	280.46	0.12	0.19
-0.10	0.00	0.00	-0.69	-0.22	0.59	0.00	279.68	0.19	0.51	8.00	-131.84	281.17	0.82	0.13
-0.07	0.00	0.00	-0.68	-0.12	0.59	-0.19	280.61	0.20	0.50	9.00	-131.31	282.49	2.14	0.07
-0.13	-0.03	0.00	-0.74	0.00	0.50	0.00	281.17	0.19	0.51	8.00	-132.59	282.66	2.31	0.06
-0.14	0.00	0.01	-0.73	0.00	0.51	0.00	281.19	0.19	0.51	8.00	-132.60	282.68	2.34	0.06

Supplemental Table 4 – Mixed effects model results for human impacts analysis.

N supply

Int.	Ag land	MPA size	Pop Den	Reef Area	Protection	AIC	R^2 fixed	R^2 cond.	df	logLik	AICc	delta	weight
0.26	0.00	0.00	-0.80	0.00	0.00	95.76	0.15	0.63	5.00	-42.88	97.58	0.00	0.42
0.11	0.00	0.00	-0.66	0.00	1.00	96.60	0.17	0.60	6.00	-42.30	99.23	1.65	0.18
0.28	0.28	0.00	-1.00	0.00	0.00	96.89	0.16	0.64	6.00	-42.45	99.52	1.94	0.16
0.31	0.00	0.00	-0.71	0.25	0.00	97.27	0.16	0.66	6.00	-42.64	99.90	2.32	0.13
0.26	0.00	0.13	-0.71	0.00	0.00	97.57	0.16	0.61	6.00	-42.79	100.20	2.62	0.11

P supply

Int.	Ag land	MPA size	Pop Den	Reef Area	Protection	AIC	R^2 fixed	R^2 cond.	df	logLik	AICc	delta	weight
0.17	0.00	0.00	-0.63	0.00	1.00	100.52	0.21	0.55	6.00	-44.26	103.14	0.00	0.25
0.43	0.00	0.00	-0.86	0.00	0.00	101.69	0.17	0.57	5.00	-45.84	103.51	0.37	0.20
0.15	0.44	0.00	-0.93	0.00	1.00	100.67	0.24	0.57	7.00	-43.34	104.29	1.15	0.14
-0.08	0.00	0.00	0.00	0.00	1.00	103.32	0.18	0.37	5.00	-46.66	105.14	2.00	0.09
0.44	0.31	0.00	-1.09	0.00	0.00	102.78	0.18	0.58	6.00	-45.39	105.40	2.26	0.08
0.11	0.00	-0.26	-0.74	0.00	1.00	102.10	0.22	0.55	7.00	-44.05	105.72	2.58	0.07
0.42	0.00	0.20	-0.72	0.00	0.00	103.31	0.17	0.55	6.00	-45.66	105.94	2.80	0.06
0.21	0.00	0.00	-0.58	0.14	1.00	102.37	0.21	0.57	7.00	-44.19	105.98	2.84	0.06
0.47	0.00	0.00	-0.79	0.17	0.00	103.48	0.17	0.59	6.00	-45.74	106.10	2.96	0.06

NP supply

Int.	Ag land	MPA size	Pop Den	Reef Area	Protection	AIC	R^2 fixed	R^2 cond.	df	logLik	AICc	delta	weight
-0.55	0.00	0.00	0.00	0.00	1.00	109.81	0.02	0.83	5.00	-49.90	111.63	0.00	0.24
-0.79	0.00	0.00	0.00	0.00	0.00	110.58	0.00	0.83	4.00	-51.29	111.76	0.13	0.22
-0.42	0.00	0.31	0.00	0.00	1.00	111.03	0.02	0.83	6.00	-49.52	113.66	2.03	0.09
-0.79	0.00	-0.15	0.00	0.00	0.00	112.25	0.00	0.83	5.00	-51.12	114.06	2.44	0.07
-0.52	-0.16	0.00	0.00	0.00	1.00	111.45	0.02	0.84	6.00	-49.73	114.08	2.45	0.07
-0.81	0.00	0.00	0.00	-0.17	0.00	112.32	0.00	0.83	5.00	-51.16	114.14	2.51	0.07
-0.78	0.00	0.00	0.11	0.00	0.00	112.41	0.00	0.82	5.00	-51.20	114.22	2.60	0.06
-0.53	0.00	0.00	-0.10	0.00	1.00	111.68	0.02	0.84	6.00	-49.84	114.30	2.68	0.06
-0.78	0.05	0.00	0.00	0.00	0.00	112.54	0.00	0.82	5.00	-51.27	114.35	2.73	0.06
-0.57	0.00	0.00	0.00	-0.08	1.00	111.75	0.02	0.83	6.00	-49.88	114.38	2.75	0.06

N storage

Int.	Ag land	MPA size	Pop Den	Reef Area	Protection	AIC	R^2 fixed	R^2 cond.	df	logLik	AICc	delta	weight
0.07	0.00	0.00	-0.52	0.00	1.00	96.37	0.19	0.55	6.00	-42.19	99.00	0.00	0.20
-0.13	0.00	0.00	0.00	0.00	1.00	97.72	0.16	0.44	5.00	-43.86	99.53	0.54	0.15
0.34	0.00	0.00	-0.75	0.00	0.00	97.86	0.14	0.57	5.00	-43.93	99.68	0.68	0.14
0.26	0.00	0.70	0.00	0.00	0.00	98.40	0.14	0.49	5.00	-44.20	100.22	1.22	0.11
0.03	0.00	0.00	0.00	0.47	1.00	98.14	0.17	0.57	6.00	-43.07	100.77	1.77	0.08
0.33	0.00	0.40	-0.48	0.00	0.00	98.16	0.16	0.57	6.00	-43.08	100.78	1.78	0.08
-0.02	0.00	0.35	0.00	0.00	1.00	98.62	0.18	0.47	6.00	-43.31	101.25	2.25	0.07
0.05	0.26	0.00	-0.69	0.00	1.00	97.68	0.20	0.57	7.00	-41.84	101.29	2.30	0.06
0.12	0.00	0.00	-0.44	0.24	1.00	97.93	0.19	0.61	7.00	-41.97	101.54	2.55	0.06
0.09	0.00	0.07	-0.49	0.00	1.00	98.34	0.19	0.56	7.00	-42.17	101.95	2.95	0.05

P storage

Int.	Ag land	MPA size	Pop Den	Reef Area	Protection	AIC	R^2 fixed	R^2 cond.	df	logLik	AICc	delta	weight
0.05	0.00	0.00	-0.53	0.00	1.00	96.79	0.19	0.55	6.00	-42.40	99.42	0.00	0.18
0.32	0.00	0.00	-0.76	0.00	0.00	98.11	0.15	0.57	5.00	-44.06	99.93	0.51	0.14
-0.13	0.00	0.00	0.00	0.00	1.00	98.24	0.16	0.43	5.00	-44.12	100.06	0.64	0.13
0.24	0.00	0.72	0.00	0.00	0.00	98.24	0.15	0.49	5.00	-44.12	100.06	0.64	0.13
0.30	0.00	0.43	-0.47	0.00	0.00	98.15	0.17	0.57	6.00	-43.08	100.78	1.36	0.09
0.01	0.00	0.00	0.00	0.49	1.00	98.45	0.18	0.58	6.00	-43.23	101.08	1.66	0.08
0.00	0.00	0.41	0.00	0.00	1.00	98.76	0.18	0.47	6.00	-43.38	101.38	1.97	0.07
0.04	0.27	0.00	-0.71	0.00	1.00	98.03	0.20	0.57	7.00	-42.02	101.64	2.23	0.06
0.11	0.00	0.00	-0.44	0.27	1.00	98.25	0.19	0.61	7.00	-42.13	101.87	2.45	0.05
0.37	0.00	0.00	-0.67	0.25	0.00	99.65	0.16	0.61	6.00	-43.83	102.28	2.86	0.04
0.08	0.00	0.13	-0.47	0.00	1.00	98.68	0.19	0.55	7.00	-42.34	102.29	2.87	0.04

C storage

Int.	Ag land	MPA size	Pop Den	Reef Area	Protection	AIC	R^2 fixed	R^2 cond.	df	logLik	AICc	delta	weight
0.07	0.00	0.00	-0.53	0.00	1.00	97.35	0.19	0.55	6.00	-42.68	99.98	0.00	0.18
0.34	0.00	0.00	-0.75	0.00	0.00	98.58	0.14	0.56	5.00	-44.29	100.40	0.42	0.15
-0.13	0.00	0.00	0.00	0.00	1.00	98.70	0.16	0.42	5.00	-44.35	100.52	0.54	0.14
0.26	0.00	0.71	0.00	0.00	0.00	99.09	0.14	0.48	5.00	-44.55	100.91	0.93	0.11
0.33	0.00	0.40	-0.48	0.00	0.00	98.88	0.16	0.56	6.00	-43.44	101.50	1.53	0.08
0.04	0.00	0.00	0.00	0.49	1.00	99.06	0.17	0.57	6.00	-43.53	101.68	1.70	0.08
0.00	0.00	0.38	0.00	0.00	1.00	99.54	0.17	0.46	6.00	-43.77	102.17	2.19	0.06
0.06	0.27	0.00	-0.71	0.00	1.00	98.61	0.20	0.56	7.00	-42.31	102.22	2.24	0.06
0.13	0.00	0.00	-0.44	0.26	1.00	98.86	0.19	0.60	7.00	-42.43	102.48	2.50	0.05
0.39	0.00	0.00	-0.66	0.25	0.00	100.14	0.15	0.61	6.00	-44.07	102.77	2.79	0.04
0.10	0.00	0.09	-0.49	0.00	1.00	99.30	0.19	0.55	7.00	-42.65	102.91	2.93	0.04

M

Int.	Ag land	MPA size	Pop Den	Reef Area	Protection	AIC	R^2 fixed	R^2 cond.	df	logLik	AICc	delta	weight
0.05	0.00	0.00	-0.55	0.00	1.00	98.39	0.21	0.53	6.00	-43.19	101.01	0.00	0.19
0.33	0.00	0.00	-0.79	0.00	0.00	99.67	0.16	0.55	5.00	-44.83	101.49	0.48	0.15
-0.18	0.00	0.00	0.00	0.00	1.00	100.01	0.18	0.40	5.00	-45.00	101.83	0.82	0.13
0.25	0.00	0.74	0.00	0.00	0.00	100.44	0.15	0.45	5.00	-45.22	102.25	1.24	0.10
0.31	0.00	0.41	-0.51	0.00	0.00	100.00	0.18	0.54	6.00	-44.00	102.63	1.61	0.09
0.04	0.37	0.00	-0.80	0.00	1.00	99.05	0.22	0.55	7.00	-42.52	102.66	1.65	0.08
0.00	0.00	0.40	0.00	0.00	1.00	100.83	0.19	0.44	6.00	-44.42	103.46	2.44	0.06
-0.01	0.00	0.00	0.00	0.42	1.00	100.91	0.18	0.51	6.00	-44.45	103.53	2.52	0.05
0.34	0.24	0.00	-0.96	0.00	0.00	101.12	0.16	0.57	6.00	-44.56	103.74	2.73	0.05
0.09	0.00	0.00	-0.50	0.16	1.00	100.20	0.20	0.56	7.00	-43.10	103.81	2.80	0.05
0.08	0.00	0.08	-0.52	0.00	1.00	100.34	0.21	0.53	7.00	-43.17	103.95	2.94	0.04

Supplemental Table 5 – Bootstrapped mixed effects model results for TL, Richness and Biomass, and Human Density (continuous measure of fishing pressure; top) and vs. Protection status (categorical variable of fishing protections, binary, fished or not fished; bottom)

Bootstrapped estimates

	Intercept	PopDensity	R^2	df	logLik
**TL**	0.30	-0.93 (0.07)	0.47	5.00	-50.42
**Biomass**	0.27	-0.70 (0.05)	0.46	5.00	-47.10
**Richness**	0.10	-0.66 (0.05)	0.34	5.00	-51.87

	Intercept	Protection	R^2	df	logLik
**TL**	-0.35	0.91 (0.07)	0.21	5.00	-50.30
**Biomass**	-0.18	0.76 (0.06)	0.21	5.00	-46.66
**Richness**	-0.29	0.72 (0.08)	0.35	5.00	-51.22

Supplemental Table 6 – Table of richness differences among trophic groups due to fishing pressure. Mixed Model column indicates results from models that include random effects for reef type and country (intercept). T-test indicates results from a standard T-test. “S” indicates 95% CIs did not overlap zero, “NS” indicates 95% CIs did overlap zero.

	Fishing	No Fishing	Sign. Mixed Model	Sign T-test
Whole Community	38.53	44.57	S	S
Whole Community - Piscivore	36.53	40.47	NS	NS
Whole Community - P and PI	31.62	33.44	NS	NS
Picivore	2.00	4.10	S	S
Picivore-Invertivore	4.90	7.03	S	S
Macroinvertivore	11.17	10.79	NS	NS
Microinvertivore	3.18	3.66	NS	NS
Planktivore	2.03	2.54	S	S
Herbivore	10.23	10.63	NS	NS
Omnivore	5.02	5.82	NS	NS

Supplemental Methods

Surveys

All sites were on fore-reef environments 10-15m deep, usually dominated by the coral Orbicella spp. Each sub-region included sites inside and outside marine reserves (i.e., no-take areas were fishing was prohibited), except at Dry Tortugas where only a reserve site was surveyed (Fig. 1, table 1). Site selection was generally haphazard: 35 of the 39 sites were selected either haphazardly or effectively randomly. The exceptions were the four sites in 'Jardines de la Reina' marine reserve, Cuba (Fig. 1, table 1) that were chosen *a priori* because there were reputed to have relatively high predator biomass [1]. In this way, the sites sampled were representative of fairly intact fish communities as well as heavily fished communities. To minimize seasonal variability, we conducted all surveys May-July in 2010-2012.

Underwater visual censuses, modified from Lang et al. 2010 [2], were used to characterize the fish assemblages. At each site we randomly placed six to eight belt transect sets parallel to the spur-and-groove habitat or along the reef slope formation following constant isobaths. In each transect we recorded fish species, abundance and estimated body size. Fish total length (TL) was binned by 10 cm class size except for individuals <10 cm TL where 5 cm interval was used; it is acknowledged that using size bins may overestimate the biomass of large fish, but this method was determined most accurate after repeatability tests conducted by the surveyers. Two divers swam along a 50 x 10 m transect as it was positioned. The first diver counted fish of medium size (5-40 cm TL) in a 30 x 2 m belt area, followed by a 15 x 1 m belt to estimate small fish <5 cm TL. The second diver counted fish > 40 cm TL within a 50 x 10 m belt to minimize bias on overestimating more mobile and large-bodied fish. The two smaller transects were contained within the largest transect to create a belt transect set. Each transect set was surveyed

in ~15 minutes, covered the entire visible water column and were at least 10 m apart. All surveys were conducted from 0800 to 1600hr. Experienced divers performed all fish surveys and fish size estimations were corroborated underwater against known size lengths. No attempt was made to survey fish behavior, a factor that could influence our surveys across the gradient of fishing pressure.

The biomass for each fish species was calculated through the allometric length-weight conversion formula, $W = aTL^b$, where W is body mass in grams, TL is the total length of each fish in cm estimated from visual surveys, and the parameters a and b are species-specific selected from geographical areas close to our study region [3] or determined from personally collected data. To calculate biomass we used the mid-point of the 5 or 10 cm interval estimates. For all the analysis we used fish biomass because it combines abundance and size resulting in a comprehensive indicator of fish assemblages status across disturbance gradients [1].

Fish species were assigned to one of seven functional groups: piscivores, piscivore-invertivores, macroinvertivores, microinvertivores, planktivores, omnivores and herbivores following reported dietary information [3]. For the purpose of this study we considered "fish predators", piscivores and/or piscivore-invertivores because both feed on fish. Invertivores (macro- and micro-) only feed on invertebrates; and omnivores mostly consume marine plants and invertebrates.

Models for nutrient excretion

The excretion and storage models allow processes of C, N and P storage and N and P supply to be estimated as a function of wet weight for 160 fish species. The analysis consisted of two steps:

1) Model all processes onto every fish in the dataset.
2) Quantify aggregate processes for each community and apply these data to hierarchical models.

Bayesian statistics allow parameters to be estimated based on observed distributions (the observed data), and prior distributions that allow knowledge from previous studies to be applied explicitly and quantitatively [4]. In this study, we used Bayesian statistics to develop models that predict excretion rate as a function of wet mass by informing empirical data (the observed data) with bioenergetics models (used to generate the priors), thus incorporating the two most widely applied methods to estimate fish excretion [5,6] into singular models of nutrient excretion by fishes. The modeling approach was developed such that if the empirical data were robust then the final model would primarily reflect these data (i.e., the priors developed from the bioenergetics model would only minimally inform the output). When the empirical data were not robust, due to lack of individual empirical measurements on rare species or high variability in the data, the final model would then be more of a reflection of the bioenergetics models (i.e., the priors would have more influence on the output). In doing so, this approach allowed us to: (1) underpin extensive empirical data to produce robust models with realistic error, and (2) fill gaps in the empirical dataset for which empirical data was incomplete.

This modeling process consisted of four steps:

(1) Bioenergetics models were developed for each family (and in some cases at the genus-level) in our dataset to estimate excretion rates of N and P for a given mass of an individual fish.

(2) These data were run in an initial Bayesian simple linear regression analysis (using uninformative priors), to generate parameter estimates for the slope and intercept

of each model ($y = mx + b$, where y is excretion rate, x is the wet mass of an individual, m is the slope and b is the intercept) (see detailed methods for bioenergetics models below).

(3) A second Bayesian simple linear regression analysis was conducted using the empirical data. In this case we used the normal posterior distributions (i.e., the mean and standard deviation) for the slope and intercept generated in *Step 2* as the priors for the model [4]. In this way we were able to take advantage of all available data and multiple approaches to generate robust estimates of nutrient supply by fishes.

(4) The posterior distributions of these final estimates for the slope and intercept were then used to calculate the excretion rate for every fish within our survey dataset – see *Ecosystem Modeling* section for further explanation.

Bioenergetics models

Bioenergetics models use a mass balance approach given *a priori* knowledge of the natural history (e.g., diet, feeding activity), physiology (e.g., stoichiometry of predator and prey, assimilation efficiency of nutrients, consumption rates, energy density of prey) and environmental conditions (temperature) to estimate nutrient excretion [5,7]. We followed the approach to construct bioenergetics models, and used diet stoichiometry data [8,9]. In total, bioenergetic models for 31 genus and 25 families within our surveys were developed.

Stoichiometry data for each family/genera was determined by averaging the percent nutrient content for a suite of species within the given level of classification. Use of parameters for closely related species may increase error in model estimates [10,11]; however, empirical work suggests that variation in excretion rates vary little within families but widely among

families[12,13]. Energy densities of prey items were obtained from [14]. Assimilation efficiencies, which have been shown to have only marginal influence on model estimates [15] were assumed to be 80% for P and 70% for N based on literature recommendations [10,11]. The growth rate of an animal has been shown to be a particularly influential parameter in bioenergetics[14], as such published growth rate values were found for each taxon of interest. Other parameter estimates were obtained from literature values specific to the given taxonomic level. Dietary parameters were determined from diet data collected by the authors [16–19] and from other published data [20].

We used Fish Bioenergetics 3.0 software [7] to determine consumption rates for the dominant feeding guilds: piscivores, piscivore-invertivores, macroinvertivores, microinvertivores, planktivores, omnivores and herbivores. To do this we chose the taxon per feeding guild for which we had the best parameter estimates (e.g., Lutjanidae >100 individuals, thousands of diet data, etc.) and used the software to calculate consumption rates based on energetic demands of the taxon. These consumption rates were then used for all families within that particular guild, holding this parameter constant and allowing other important estimates (e.g., body stoichiometry, prey stoichiometry and growth rate) to have influence over the model. Bioenergetics models were run using R software [21].

To account for inherent error that occurs when parameterizing such models, we propagated uncertainty associated with diet content and consumption rates, two parameters that are highly influential in bioenergetics models [5,7,22], through the models using Monte Carlo simulations. Specifically, a normal distribution of values was created for each parameter with a standard deviation of 5% of the maximum potential value of that parameter (in both cases the parameters represent a proportion, so the standard deviation was 0.05). For each model run, random draws were taken from within these distributions 500-10,000 times, depending on the

size range of the fish within that family. Note the number of draws within this range did not change the outcome of the model.

Empirical excretion estimates

All fish were captured using hook and line or traps on Abaco Island, The Bahamas between 2008-2011. Fish were captured in coral reef, mangrove and seagrass ecosystem types. Fish were pooled across ecosystem type, such that individuals from a given species could have been caught in any one or all ecosystems. We accounted for potential differences in resource availability across ecosystem type, which would be predicted to affect recycling rates, in two ways: (1) individuals within a given species were often collected from different ecosystem types and potential variation across ecosystem type was pooled, and thus accounted for, in our empirical models, (2) we modeled error for diet nutrient content in our bioenergetics models. Excretion rates, for nitrogen - NH_4^+ and phosphorus – soluble reactive phosphorus (SRP), were measured *in situ* following the methodologies of [23] , as modified by [6]. Values were control corrected through the use of multiple (typically n=6) identical control incubation bags without fish. Each fish used for excretion experiments (n = 665 individual fish, 79 species, 46 genera and 26 families; size range: 2-107 cm) was weighed for wet mass and measured to standard length. Fish were identified, and dissected to remove stomach contents, and then frozen for transport to the University of Georgia's (UGA) Odum School of Ecology and processed for elemental content (C, N and P). UGA's Institutional Animal Care and Use Committee approved protocols for the capture and handling of fish (AUP # A2009-10003-0) were used. Water samples (filtered with 0.45 μm Whatman nylon membrane filters) were immediately placed on ice and, within 10 hours, analyzed for NH_4 using the methodologies of [24], or frozen for transport to UGA for SRP analyses using the ascorbic acid method and colorimetric analyses [25].

Bayesian excretion models

Previous research on fish nutrient stoichiometry has shown that variation within families is relatively constrained [12]. As such, we used genus- or family-level bioenergetics models to inform empirical data in a Bayesian framework (i.e., bioenergetics models were employed to constrain excess variance in empirical excretion models when present). To further illustrate this approach, we follow each step taken to generate the final equation (*excretion rate = wet mass*slope + intercept*) (provided above) with an example species: gray snapper (*Lutjanus griseus*). **Step 1:** A genus-level bioenergetics model for *Lutjanus* was developed. **Step 2:** A Bayesian simple linear analyses was run using the size-specific data generated from the bioenergetics model. **Step 3:** A second Bayesian simple linear analysis was run using the empirical excretion data (i.e., collected from individual gray snapper; n=70), whereby the priors for this model were determined by the estimates for the slope and intercept calculated from the bioenergetics data in Step 2. **Step 4:** The estimates (and standard deviation) for the slope and intercept from this final model were applied to calculate the excretion rate for all gray snapper found within the dataset using Monte Carlo simulations (see *Ecosystem Modeling* section below).

In the case of the gray snapper models, the empirical data were robust and thus the priors generated from the bioenergetics model had little influence on the final model (e.g., the slope from the empirical data alone, the bioenergetics model, and the final model were: $m_{emprical}$ = 0.000018, $m_{bioenergetic}$ = 0.0001, m_{final} = 0.000022). In cases where the empirical data was less robust, the bioenergetics model would have more influence on the final slope estimate. In all cases the prior estimates influenced the variance associated with each parameter estimate. To account for all potential sources of error we used Monte Carlo simulations to perpetuate

uncertainty into our final estimates of excretion for each individual fish (see *Ecosystem modeling* below).

All models were constructed at the level of taxonomic resolution for which we had optimal data. For example, if there were not sufficient empirical data at the species level to generate significant linear models of wet mass vs. excretion (at $\alpha = 0.1$, typically >8 individuals), data would be pooled among species within the same genus and informed with the appropriate genus-level bioenergetics model. Using this approach, we developed 27 species-, 25 genus- and 16 family-level models. With these models nutrient supply and storage could be estimated for 144 of the 158 species. Using this approach we accounted for 99.4% of the biomass of fishes within the field surveys.

All models were run with three chains for 50,000 iterations with a burn-in period of 1000. Data for excretion models were not transformed and assumptions of normality were met. Bayesian analysis was run using the "rjags" package in R [21].

It is important to note that, as is the case with all models, there are potential biases that could influence our estimated excretion rates of coral reef fishes. First, empirical estimates have three primary potential issues: the stress on fish due to capture and handling, and oxygen deprivation that can increase nutrient excretion rates, and starvation, that can reduce excretion rates [6]. We attempted to account for these issues following the recommendations of [6], as well as conducting our own time trial experiments to determine the most appropriate time to take measurements such that the influence of handling stress, oxygen stress, and starvation is minimized (see [13]). The primary bias associated with our bioenergetics models is the lack of parameter estimates for each of the families within our dataset. It is for these reasons that we used a Bayesian framework to allow the bioenergetics models to inform the empirical data.

While we believe this approach to be a robust alternative to using solely empirical or bioenergetics models, we nonetheless recognize its limitations. To further account for error in model estimates, we use error propagation when scaling individual rates of excretion to entire fish communities. However, it is recognized that utility of error propagation is nonetheless still limited by any potential systematic bias in the initial model.

Ecosystem modeling

Using the equations generated from the Bayesian models, excretion estimates were modeled onto each individual fish (n= 72,824) in 43 fore-reefs (10-15 m deep) in five Caribbean countries surveyed for multiple years (110 surveys total). This was used to quantify species-level and then aggregate community-level rates of N and P supply and storage. This included 143 fish species representing ~20% of the fish species across all ecosystem types in the Caribbean [26,26]. Fish nutrient supply is a function of body size, organism identity and diet [5,12]. As such, we used Monte Carlo simulations to model uncertainty into our estimates of fish nutrient supply for individual fish within the dataset. For each fish, we sampled from the posterior distribution of both the slope and intercept from our Bayesian excretion models to calculate 1000 mass-based species-specific excretion estimates. These values were summed to provide a distribution of community-level aggregate estimates (n=1000) of N and P supply. We applied the same methodology to calculate nutrient storage, whereas in this case we sampled 1000 times from the normal distribution (mean ± standard deviation) associated with our stoichiometric estimates for body nutrient content at each taxonomic level (typically genus or species). In doing so, we modeled realistic estimates of error into each step of our analysis to create a range of values that represent a realistic distribution of nutrient supply and storage for every fish and the entire

community.   Because we sampled from a normal distributions for each estimate, this error propagation approach should not inherently alter the mean value of the aggregate community-level excretion, but instead provide information as to how much variability there may be in this mean as a result of potential error and natural variability.

Hierarchical Mixed Effects Models

We used hierarchical mixed effects models and information theory (Akaike information criterion, AIC_c) [27] , to explore the relationship between the aggregate supply, and storage of nutrients and multifunctionality (*M*) and community assembly. To do so we ran six separate models, one for each of the ecosystem processes of interest and for *M*. All models included the same six parameters: Species Richness (Richness), Species evenness (SD - reciprocal Simpson's index)[28], Trophic Group evenness (TG), mean biomass-weighted Trophic Level (TL; trophic level was used from), mean Maximum Size of each species within the community (Max Length) calculated following [29] and skewness of the size frequency distribution of the community (Skewness)[30]. All model assumptions were met and Pearsons correlations among predictors showed that all were less than $r = 0.3$ with the exception of Richness and Species evenness in which the correlation was $r = 0.53$. Biomass has long been recognized to be a strong predictor of stoichiometric properties [31], and in the case of this study was directly used to calculate our response values. For this reason we did not include biomass as a predictor in our models. However to test for the importance of biomass we divided all responses by community level biomass to get a community-level mass-specific process, e.g., grams of N storage per grams of biomass.

Multifunctionality was calculated following [32], whereby we calculated the average of the Z scores for each ecosystem process of interest. Z scores were calculated from log transformed normalized data: Z score = $[x - \mu] / \sigma$; where x is the site-level ecosystem process, μ is the mean value for all sites, and σ is the standard deviation of all sites. This index was chosen for three primary reasons: 1) it follows a normal distribution, 2) all of our response variables were positively correlated, and 3) z scores do not constrain the variability found in the raw data [32].

All variables were calculated for each transect (mean 6.2 transects per site) and then averaged for a given survey data for a given Site (n=110 total). All 110 surveys were used for the biodiversity with Site (at which multiple surveys were conducted) nested within Country and reef types (habitat) as random intercept effects in all models to account for variability that may exists due to these factors. Models were run using the "lme4" package in R [21]. All response variables were z-score transformed and all predictors were standardized in order to make comparisons among estimates. No issues were found with collinearity among the predictor variables. In all cases, model assumptions of normality and homogeneity of variance were met.

Human Impact Analysis

We used hierarchical mixed effects models and information theory (Akaike information criterion, AIC_c) [27], to explore the relationship between the aggregate supply, and storage of nutrients and multifunctionality (*M*) and attributes of human impacts (below). Model structure was similar to the biodiversity analyses (above), however here we averaged surveys across years within a given site, resulting in 43 total sites (response variables). For these models, Country was nested within reef type as the random intercept in all models to account for variability that may exists due to these factors. Models were run using the "lme4" package in R [21]. All response

variables were z-score transformed and all predictors were standardized in order to make comparisons among estimates. No issues were found with collinearity among the predictor variables. In all cases, model assumptions of normality and homogeneity of variance were met.

For each reef site, we gathered a comprehensive data set of anthropogenic and environmental variables (table 2). Fishing pressure could not be accurately estimated for each surveyed site because of incomplete information on fishing activities. Thus, we assumed that human population parameters, e.g., distance to nearest population centers - indicator of long-distance effects, calculated from each site to the center of population settlements, were an adequate measurement of anthropogenic impacts (i.e. harvesting intensity) as the number of people has been positively correlated with fishing pressure in the Caribbean [33–36]. Projection estimates of human population counts for the year 2010 were obtained from the Gridded Population of the World V.3 at 0.25 degree resolution [37] and calculated in ArcGIS v10.0.

Anthropogenic impacts per site were measured as: (1) area of cultivated land within 50 km, a proxy of terrestrial runoff (Agriculture), and (2) distance to the nearest population settlement (Population Density). We also measured marine reserve size, reserve age and poaching level (low or high), and the total area of the reef tract within 5km of the survey area.

Mixed effects models were used (with random intercepts on reef type and country) to assess the relative influence of fishing pressure (both as a continuous variable of population density and as a categorical variable of fished and protected reefs) on TL, Richness and Biomass. Bootstrapped regressions (n=100) were used to generate estimates of the slope for these relationships. In all cases response variables were z-scored so relative effect sizes could be compared directly.

Distributions of effect sizes used to illustrate the relative degree to which fishing reduces the supply and storage of nutrients by different trophic groups (see Fig. 3) were calculated through the use of a bootstrap procedure. The effect size here represents the percent reduction between reef communities that are fished and those that are not fished.

Data Availability

The authors declare that the primary data supporting the findings of this study are available within the article and its supplementary information files.

Supplemental References

1. Newman, M. J. H., Paredes, G. A., Sala, E. & Jackson, J. B. C. Structure of Caribbean coral reef communities across a large gradient of fish biomass. *Ecol. Lett.* **9,** 1216–1227 (2006).
2. Lang, J. C., Marks, K. W., Kramer, P. A., Richards Kramer, P. & Ginsburg, R. N. AGRRA Protocols version 5.4. (2010).
3. Froese, R. & Pauly, D. FishBase. *World Wide Web Electron. Publ.* (2012).
4. McCarthy, M. A. *Bayesian Methods for Ecology*. (Cambridge University Press, 2007).
5. Schreck, C. B. & Moyle, P. B. *Methods for fish biology*. (American Fisheries Society, 1990).
6. Whiles, M. R., Huryn, A. D., Taylor, B. W. & Reeve, J. D. Influence of handling stress and fasting on estimates of ammonium excretion by tadpoles and fish: recommendations for designing excretion experiments. *Limnol. Oceanogr.* **7,** 1–7 (2009).
7. Hanson, P. C., Johnson, T. B., Schindle, D. E. & Kitchell, J. F. *Fish Bioenergetics 3.0*. (University of Wisconsin System Sea Grant Institute, 1997).
8. Allgeier, J. E., Layman, C. A., Mumby, P. J. & Rosemond, A. D. Consistent nutrient storage and supply mediated by diverse fish communities in coral reef ecosystems. *Glob. Chang. Biol.* **20,** 2459–2472 (2014).
9. Allgeier, J. E., Layman, C. A., Mumby, P. J. & Rosemond, A. D. Biogeochemical implications of biodiversity loss across regional gradients of coastal marine ecosystems. *Ecol. Monogr.* **85,** 117:132 (2015).
10. Hansen, M. J. *et al.* Applications of bioenergetics models to fish ecology and management - where do we go from here? *Trans. Am. Fish. Soc.* **122,** 1019–1030 (1993).

11. Ney, J. J. Bioenergetics modeling today - growing pains on the cutting edge. *Trans. Am. Fish. Soc.* **122,** 736–748 (1993).

12. Vanni, M. J., Flecker, A. S., Hood, J. M. & Headworth, J. L. Stoichiometry of nutrient recycling by vertebrates in a tropical stream: linking species identity and ecosystem processes. *Ecol. Lett.* **5,** 285–293 (2002).

13. Allgeier, J. E., Wenger, S. J., Schindle, D. E., Rosemond, A. D. & Layman, C. A. Metabolic theory and taxonomic identity predict nutrient cycling in a diverse food web. *Proc. Natl. Acad. Sci.* **112,** 2640–2647 (2015).

14. Cummins, K. W. & Wuycheck, J. C. *Caloric Equivelances for Investigations in Ecological Energetics*. (1981).

15. Hood, J. M., Vanni, M. J. & Flecker, A. S. Nutrient recycling by two phosphorus-rich grazing catfish: the potential for phosphorus-limitation of fish growth. *Oecologia* **146,** 247–257 (2005).

16. Layman, C. A. & Silliman, B. R. Preliminary survey and diet analysis of juvenile fishes of an estuarine creek on Andros Island, Bahamas. *Bull. Mar. Sci.* **70,** 199–210 (2002).

17. Layman, C. A., Quattrochi, J. P., Peyer, C. M. & Allgeier, J. E. Niche width collapse in a resilient top predator following ecosystem fragmentation. *Ecol. Lett.* **10,** 937–944 (2007).

18. Hammerschlag-Peyer, C. M. & Layman, C. A. Intrapopulation variation in habitat use by two abundant coastal fish species. *Mar. Ecol. Ser.* **415,** 211–220 (2010).

19. Layman, C. A. & Allgeier, J. E. Characterizing trophic ecology of generalist consumers: a case study on the invasive Lionfish Pterois volitans in the Bahamas. *Mar. Ecol. Prog. Ser.* **448,** 131–144 (2012).

20. Munro, J. L. *Caribbean Coral Reef Fisheries Resouces*. **2,** (International Center for Living Aquatic Resouces Management, 1983).

21. R Core Development Team. R: A language and environment for statistical computing. *R Found. Stat. Comput.* (2012). at <http://www.r-project.org>

22. Schindler, D. E. & Eby, L. A. Stoichiometry of fishes and their prey: Implications for nutrient recycling. *Ecology* **78,** 1816–1831 (1997).

23. Schaus, M. H. *et al.* Nitrogen and phosphorous excretion by detritivorous gizzard shad in a reservoir ecosystem. *Limnol. Oceanogr.* **42,** 1386–1397 (1997).

24. Taylor, B. W. *et al.* Improving the fluorometric ammonium method: matrix effects, background fluorescence, and standard additions. *J. North Am. Benthol. Soc.* **26,** 167–177 (2007).

25. APHA. Standard Methods for the Examination of Water and Wastewater. American Public Health Association, American Water Works Association, and Water Pollution Control Federation. (1995).

26. Spalding, M., Ravilious, C. & Green, E. *World Atlas of Coral Reefs*. (California Press, 2001).

27. Burnham, K. P. & Anderson, D. R. *Model selection and multimodel inference: a practical information theoretic approach*. (Springer-Verlag, 2002).

28. Simpson, E. H. Measurement of diversity. *Nature* **163,** 688 (1949).

29. Nicholson, M. D. & Jennings, S. Testing candidate indicators to support ecosystem-based management: the power of monitoring surveys to detect temporal trends in fish community metrics. *Ices J. Mar. Sci.* **61,** 35–42 (2004).

30. Joanes, D. N. & Gill, C. A. Comparing measures of sample skewness and kurtosis. *J. R. Stat. Soc. Ser. D-the Stat.* **47,** 183–189 (1998).

31. Sterner, R. W. & Elser, J. J. *Ecological Stoichiometry: The Biology of Elements from Molecules to the Biosphere*. (Princton University Press, 2002).

32. Maestre, F. T. *et al.* Plant Species Richness and Ecosystem Multifunctionality in Global Drylands. *Science (80-.).* **335,** 214–218 (2012).

33. Newton, K., Côté, I. M., Pilling, G. M., Jennings, S. & Dulvy, N. K. Current and Future Sustainability of Island Coral Reef Fisheries. *Curr. Biol.* **17,** 655–658 (2007).

34. Stallings, C. D. Fishery-Independent Data Reveal Negative Effect of Human Population Density on Caribbean Predatory Fish Communities. *PLoS One* **4,** e5333 (2009).

35. Ward-Paige, C. A. *et al.* Large-Scale Absence of Sharks on Reefs in the Greater-Caribbean: A Footprint of Human Pressures. *PLoS One* **5,** e11968 (2010).

36. Mora, C. A clear human footprint in the coral reefs of the Caribbean. *Proc. R. Scocitey B - Biol. Sci.* **275,** 767–773 (2008).

37. SEDAC, S. D. and A. C. *Global Rural-Urban Mapping Project – settlement points.* (2010).
